# Carbon Dots from Dried German Chamomile Flower and Its Residual Biomass: Characteristics, Bioactivities, Cytotoxicity and Its Preservative Effect on the Refrigerated Precooked Baby Clam (*Paphia undulata*)

**DOI:** 10.3390/foods14173130

**Published:** 2025-09-07

**Authors:** Birinchi Bora, Suriya Palamae, Bin Zhang, Tao Yin, Jun Tae Kim, Jong-Whan Rhim, Soottawat Benjakul

**Affiliations:** 1 International Center of Excellence in Seafood Science and Innovation (ICE-SSI), Faculty of Agro-Industry, Prince of Songkla University, Hat Yai, Songkhla 90110, Thailand; 6711030001@psu.ac.th (B.B.); suriya.pal@psu.ac.th (S.P.); 2 Key Laboratory of Health Risk Factors for Seafood of Zhejiang Province, College of Food Science and Pharmacy, Zhejiang Ocean University, Zhoushan 316022, China; zhangbin_ouc@163.com; 3 College of Food Science and Technology, Huazhong Agricultural University, Wuhan 430070, China; yintao@mail.hzau.edu.cn; 4 BioNanocomposite Research Center, Department of Food and Nutrition, Kyung Hee University, 26 Kyungheedae-ro, Dongdaemun-gu, Seoul 02447, Republic of Korea; jtkim92@khu.ac.kr (J.T.K.); jwrhim@khu.ac.kr (J.-W.R.)

**Keywords:** carbon dots, agriculture waste, utilization, shelf life extension, baby clam, preservatives

## Abstract

The growing demand for natural and sustainable food preservatives has drawn interest in carbon dots (CDs) derived from plant sources. This study aimed to synthesize CDs from dried German chamomile flowers (DF) and residual biomass (RB) obtained after essential oil extraction using a hydrothermal process. Their characteristics, bioactivities and cytotoxicity were examined. Both DF-CDs and RB-CDs were spherical (7–10 nm), exhibited strong UV blocking properties and tunable fluorescence and were rich in polyphenolic functional groups, especially the –OH group. DF-CDs generally showed higher antioxidant capacity than RB-CDs as assayed by DPPH, ABTS radical scavenging activities, FRAP and metal chelation activity. Both CDs showed antibacterial effects toward pathogenic bacterial strains (*Escherichia coli* and *Listeria monocytogenes*) and spoilage bacteria (*Shewanella putrefaciens* and *Pseudomonas aeruginosa*) in a dose-dependent manner. Cytotoxicity was assessed in BJ human fibroblasts, and both CDs exhibited high biocompatibility (>88% viability at 1000 µg/mL). When both CDs at 300 and 600 ppm were applied in a precooked baby clam edible portion (PBC-EP) stored at 4 °C, microbial growth, TVB and TMA contents were lower than those of the control. The total viable count was still under the limit (5.8 log CFU/mL) for the sample treated with CDs at 600 ppm up to 9 days, while the control was kept for only 3 days. Furthermore, the lipid oxidation level (PV and TBARS value) of PBC-EP decreased with CD treatment, especially at higher concentrations (600 ppm). Therefore, chamomile-derived CDs could serve as a promising alternative for perishable seafood preservation.

## 1. Introduction

In recent years, there has been a growing emphasis on sustainable approaches to food preservation, particularly in response to increasing consumer demand for natural and environmentally friendly additives instead of synthetic counterparts [1,2,3]. The food industry has faced the challenges of ensuring product safety and minimizing quality deterioration during storage and distribution, especially for highly perishable products such as seafood [4,5]. Recently, nanotechnology and green chemistry have become innovative solutions, and the development of nanomaterials with multifunctional properties has gained tremendous interest [6,7]. Carbon dots (CDs) have appeared as a novel carbon-based nanomaterial which has significant potential in food, biomedical and environmental applications [8,9].

CDs are quasi-spherical nanoscale particles, typically with sizes less than 10 nm. They have been known for their excellent photoluminescence, biocompatibility, water solubility and surface functional properties [10,11]. These properties make them appropriate for a wide range of applications, including imaging, drug delivery, biosensing, and antimicrobial and antioxidant agents for food preservation [12,13,14]. Importantly, CDs can be synthesized from a variety of natural and organic sources, including plant materials, agricultural residues and food waste, using environmentally mild synthesis methods such as hydrothermal carbonization [15]. Overall, precursor materials significantly influence the physicochemical and functional properties of the resulting CDs, which directly affect their applicability in different fields [16].

German chamomile (*Matricaria chamomilla* L.), a widely cultivated medicinal plant, is traditionally used as an herbal remedy since it is rich in phytochemicals, including flavonoids (e.g., Apigenin), phenolic acids and essential oils [17]. These bioactive compounds not only contribute to their therapeutic properties but also serve as a valuable carbon source for the green synthesis of functional nanomaterials. Despite its widespread use in traditional medicine and the food industry [17,18], chamomile flower and its processing residues have not been exploited as precursors for CD synthesis. The utilization of plants and their waste not only aligns with the waste valorization principle but also contributes to circular bioeconomy practices, in which value-added products like CDs can be produced.

The undulated surf clam (*Paphia undulata*), commonly known as baby clam, is a commercially important bivalve mollusk found in shallow coastal waters [19]. It is commonly found along the southern coastline of Thailand, as well as southern regions of China and parts of Southeast Asian countries. In 2020, Thailand harvested approximately 2600 tonnes of baby clams, generating a market value of THB 175.4 million [20]. Baby clams are highly prized owing to their distinctive taste with good nutritional profiles [21]. Nonetheless, baby clam, even in the precooked form, undergoes spoilage easily. Therefore, potential active packaging is required to lengthen its shelf life while maintaining quality.

CDs have been used for various purposes, e.g., as antioxidant agents, antimicrobial agents, UV blockers, freshness indicators and multipurpose components. They have been used to improve the barrier and mechanical properties of biopolymer films and extend the shelf life of some foods [22,23]. However, to our knowledge, chamomile-derived CDs have not been investigated for seafood preservation.

Therefore, CDs from dried German chamomile flowers (DF) and their residual biomass (RB) obtained from essential oil extraction were synthesized using a hydrothermal method and characterized. Their antioxidant, antimicrobial and cytotoxic profiles were also evaluated. The effect of both CDs on extending the shelf life of a precooked edible portion of baby clam stored at refrigerated temperature was also investigated, which will help in addressing the unexplored application of plant-derived CDs as sustainable nanomaterials for highly perishable matrices such as seafoods.

## 2. Materials and Methods

### 2.1. Chemicals, Microbial Media and Baby Clam Samples

All chemicals employed in this study were analytical-grade. The reagents, including 2,2-diphenyl-1-picrylhydrazyl (DPPH), thiobarbituric acid (TBA), trichloroacetic acid (TCA), 2,2′-azino-bis (3-ethylbenzothiazoline-6-sulfonic acid) (ABTS), ferrous chloride, ferrozine, Trolox, cumene hydroperoxide, TPTZ (2,4,6-tris(2-pyridyl)-s-triazine), ammonium thiocyanate and ferric chloride, were sourced from Sigma-Aldrich (St. Louis, MO, USA). Microbiological media, including tryptic soy agar (TSA), tryptic soy broth (TSB), plate count agar (PCA), Mueller–Hinton Agar (MHA) and Mueller–Hinton Broth (MHB), were obtained from Himedia (Mumbai, India).

The precooked edible portion of baby clam used in this study was produced by Klamai Sealand Company Ltd., located in Mueang Surat Thani, Surat Thani province, Thailand. The sample was obtained from a supermarket in Hat Yai, Thailand, during June and July 2025 and stored at 4 °C. Each baby clam had an average weight of 1.5 ± 0.5 g and an average length of 1.3 ± 0.5 cm. Precooked baby clams obtained less than 2 days after production (1 kg) were packed in a plastic bag and transported on crushed ice from the supermarket to the laboratory within 30 min.

### 2.2. Synthesis of CDs from Dried German Chamomile Flower and Residual Biomass

Fresh German chamomile (*Matricaria chamomilla* L.) flowers were collected from Palampur, Himachal Pradesh, India, along with their spent biomass obtained after essential oil extraction [24]. The fresh flowers and residual biomass obtained after essential oil extraction were washed thoroughly with tap water and then dried in a tray dryer at 50 °C. After the samples reached a moisture content of approximately 5%, they were ground into powder and sieved using a screen with 60 mesh. The DF and RB powders were sealed in zip-lock bags and stored at 4 °C until further use.

CDs were synthesized from DF and RB powders using a hydrothermal approach. Firstly, the powder was prepared in distilled water to obtain a concentration of 2% (*w*/*v*), and the suspension was transferred into a Teflon-lined stainless-steel autoclave. The mixtures were then subjected to heat treatment in a muffle furnace at 200 °C for 6 h. After cooling to room temperature, the resulting yellowish-brown suspensions from both samples were centrifuged at 5000× *g* for 15 min and subsequently passed through a 0.20 µm membrane filter. The obtained solutions containing CDs were designated as “DF-CDs and RB-CDs” and stored at 4 °C until further analysis.

### 2.3. Characterization of CDs

#### 2.3.1. Visual Appearance and Color

The visual appearances of DF-CDs and RB-CDs under both normal light and UV light (365 nm) were recorded using a smartphone camera (Redmi 13C, 5G, Xiaomi, Beijing, China). The color changes were observed by the measurement of CIE Lab values (L* = lightness; a* = redness and greenness; b* = yellowness and blueness) using a Hunter Lab (Colorflex, Reston, VA, USA) [25].

#### 2.3.2. UV-Vis Spectrophotometric and Spectrofluorometric Spectra

The light absorption spectra of the CD samples (20–100 μg/mL) were analyzed using a UV-Vis spectrophotometer (Mecasys Optizen POP Series UV/Vis, Kyoto, Korea) over a wavelength range of 200–800 nm. Quartz cuvettes with a path length of 1 cm were used for all measurements. Before determination, the instrument was calibrated using a blank of deionized water, which also served as the reference. The CD samples were then introduced into the cuvette, and the absorbance spectra were collected at room temperature [25]. The fluorescence spectral properties of the aqueous DF-CD and RB-CD solutions (200 µg/mL) were measured using a spectrofluorometer (Hitachi, Model F-7100 FL, Kyoto, Japan) equipped with a xenon lamp as the excitation source. The spectra were acquired at 450 nm. The maximal fluorescence emission activity was attained by increasing the excitation wavelength from 200 to 750 nm with a 10 nm increase interval. The bandwidth was set at 5 nm, and the scan speed was set at 1200 nm/min [25].

#### 2.3.3. UV Blocking Property

The UV barrier performance of CD solutions (20–100 µg/mL) was evaluated following the method described by Koutchma et al. [26]. The percentage of UV-A and UV-B light blocked by the CDs was calculated using the following equations:

(1) $$UV-A\;\mathrm{blocking}\left(\%\right)=100-\frac{\int_{320}^{400}T\left(\lambda \right)d\lambda }{\int_{320}^{400}d\lambda }$$

(2) $$UV-B\;\mathrm{blocking}\left(\%\right)=100-\frac{\int_{280}^{320}T\left(\lambda \right)d\lambda }{\int_{280}^{320}d\lambda }$$

where T(λ) represents the average transmittance of the CDs at a given wavelength λ, and dλ represents the bandwidth interval at the wavelength λ.

#### 2.3.4. TEM and Particle Size

The morphology of the CD samples was examined using a transmission electron microscope (TEM) with a field-emission TEM instrument (Talos F200i model, Thermo Scientific Co., Ltd., Waltham, MA, USA.) operating at 200 kV. CDs were dispersed in water at a ratio of 1:2 (*w*/*v*). Grids were prepared by pipetting 3 µL of the CD samples on 300-mesh Cu (Cu-300HD) coated with holey/thin carbon films (Grid-Tech), followed by the evaporation of the solvent. Subsequently, the average particle size of the CDs was determined by calculating the average diameter of all the particles identified in the TEM images [25].

#### 2.3.5. Fourier Transform Infrared (FTIR) Spectra

The functional groups and secondary structures of freeze-dried powder samples (DF-CDs and RB-CDs) were examined using an FTIR spectrometer (Model Equinox 55, Bruker Co., Ettlinger, Germany). Measurements were carried out in the 4000–500 cm^−1^ range, with 32 scans and a resolution of 4 cm^−1^. Before the analysis, sample pellets were prepared by mixing the powder with potassium bromide at a 1:10 ratio [25].

### 2.4. Antioxidant Activity

#### 2.4.1. 2,2-Diphenyl-1-Picrylhydrazyl Radical Scavenging Activity (DPPH-RSA)

The DPPH-RSA of both DF-CDs and RB-CDs was determined as described by Wu et al. [27]. CDs were diluted in water to obtain a concentration of 1 mg/mL. Thereafter, 150 μL of 0.15 mM DPPH dissolved in 99% ethanol was mixed with an equal volume of CD solution in a 96-well microtiter plate. The absorbance at 517 nm was measured after 30 min of incubation. Ethanol (99%) was utilized to replace the DPPH solution and considered as the blank. A standard curve of Trolox (10–50 μM) was prepared. Activity was computed after blank subtraction and reported as mmol Trolox equivalent (TE)/mL.

#### 2.4.2. 2,2′-Azino-Bis (3-Ethyl Benzothiazoline-6-Sulfonic Acid) Radical Scavenging Activity (ABTS-RSA)

ABTS-RSA was examined by combining 10 µL of CDs (1 mg/mL) with 190 µL of the ABTS working solution prepared following the method of Benzie and Strain [28]. After a 45 min incubation period, the absorbance was measured at 734 nm. ABTS-RSA was then calculated and expressed as mmol Trolox equivalents (TE)/mL.

#### 2.4.3. Ferric Reducing Antioxidant Power (FRAP)

The FRAP assay was performed by combining 10 µL of the sample with 190 µL of FRAP reagent, followed by incubation for 30 min. The absorbance was then measured at 593 nm. Antioxidant activity was assessed, based on the reduction of the ferric–TPTZ complex to its ferrous form, and expressed as mmol Trolox equivalents (TE)/mL [29].

#### 2.4.4. Metal Chelating Activity (MCA)

MCA was assessed by combining 940 µL of the CD samples with 20 µL of 2 mM ferrous chloride and 40 µL of 5 mM ferrozine. A blank was prepared similarly, except that distilled water was used to replace the ferrous chloride solution. The absorbance was measured at 562 nm. MCA was reported as µmol EDTA equivalent/mL [29].

### 2.5. Antimicrobial Activity

#### 2.5.1. Bacterial Strains, Culture Conditions and Cell Suspension

The bacterial strains used included *Shewanella putrefaciens* (JCM 20190), sourced from the Japan Collection of Microorganisms at the RIKEN Bioresource Research Centre in Ibaraki, Japan; *Pseudomonas aeruginosa* (ATCC 27853) and *Escherichia coli* (ATCC 25923), which were gifted by the American Type Culture Collection (ATCC Manassas, VA, USA); and *Listeria monocytogenes* (FSL J1 208) was sourced from the Food Safety Laboratory (FSL) at Cornell University, Ithaca, NY, USA. Each strain was individually cultured in tryptic soy broth (TSB) for 24 h. Following this, the bacterial cells were resuspended in fresh TSB and incubated at 37 °C for an additional 4 h. The resulting bacterial suspensions were then serially diluted to achieve a concentration of 10^8^ CFU/mL, corresponding to a 0.5 McFarland standard.

#### 2.5.2. Minimum Inhibitory Concentration (MIC) and Minimum Bactericidal Concentration (MBC)

To determine the MIC and MBC, the procedure described by Tariq et al. [30] was adopted. DF-CDs and RB-CDs were serially diluted (ranging from 32.77 to 0.064 mg/mL) in a sterile 96-well microplate. Each well was then inoculated with 100 µL of bacterial suspension (10^6^ CFU/mL) and incubated at 37 °C for 24 h. The MIC was identified as the lowest concentration that completely inhibited bacterial growth. After incubation, 50 µL of resazurin solution (2 mM) was added and incubated for an additional 1–2 h at 37 °C, and the color change from purple to pink was observed visually. The MBC was defined as the lowest concentration that completely eradicated the bacterial isolates when determined using the spot plate method. A total of 10 µL of aliquots from wells with no visible bacterial growth was plated on tryptic soy agar (TSA) plates.

#### 2.5.3. Bacterial Growth and Biofilm Formation

Biofilm formation was assessed according to the method described by Sharma et al. [31]. Inocula of *Shewanella putrefaciens*, *Pseudomonas aeruginosa*, *Escherichia coli* and *Listeria monocytogenes* (10^6^ CFU/mL) were prepared in TSB medium and added to each well of a microtiter plate. DF-CDs and RB-CDs were introduced at the final concentrations of 2MIC, MIC, MIC/2, MIC/4 and MIC/8. A total of 100 μL of the sample and 100 μL of bacterial inoculum were transferred into a 96-well plate. The plates were incubated at 37 °C for 48 h. Bacterial growth was monitored by measuring absorbance at 600 nm. After removing the planktonic cells, the wells were washed three times with sterile phosphate-buffered solution and then air-dried for 30 min. Biofilms were stained with 0.1% crystal violet at 25 °C for 30–45 min. After staining, the wells were rinsed three times with sterile distilled water and left to air-dry overnight. To solubilize the dye, 200 μL of absolute ethanol was added to each well and incubated for 10 min. Biofilm quantity was then determined by measuring the absorbance at 570 nm.

Using the following formula, the relative biofilm formation index (BFI) was computed [32]:

BFI = A_biofilm_ 570 − A_control_ 570/A_bacteria_ 600 − A_media_ 600 (3)

where A_biofilm_ 570 = the absorbance of the crystal violet-stained biofilm formed in the wells after 48 h at 570 nm; A_control_ 570 = the absorbance of the blank wells containing only TSB (control) at 570 nm; A_bacteria_ 600 = absorbance for the bacterial culture in the wells after 48 h at 600 nm; A_media_ 600 = the absorbance of TSB without bacteria (media blank) at 600 nm.

### 2.6. Cytotoxicity

BJ human foreskin fibroblast cultures were sourced from the American Type Culture Collection (ATCC; Manassas, VA, USA). Fibroblast cells were maintained in Eagle’s Minimum Essential Medium (EMEM) (Gibco, Grand Island, NY, USA) enriched with 10% Fetal bovine serum and 1% antibiotic–antimycotic at 37 °C in a 5% CO_2_ incubator (BINDER GmbH, Tuttlingen, Germany). When the cells reached 80–90% confluency after about 10–12 passages, they were detached using 0.25% trypsin–EDTA and subsequently sub-cultured.

MTT assay was employed. In living cells, MTT forms formazan, which has peak absorbance between 550 and 600 nm and correlates proportionally with cell viability. Cell Proliferation Kit I (Roche Diagnostics, Basel, Switzerland) was used following the manufacturer’s protocol. Briefly, BJ cells were plated in a 96-well plate at 1 × 10^4^ cells/well and incubated at 37 °C in a CO_2_ incubator. Following 24 h of incubation, the cells were exposed to DF-CDs and RB-CDs at varying concentrations of 125, 250, 500 and 1000 µg/mL for 24 and 48 h [33]. MTT solution (10 µL) was added to each well, and cells were further incubated for 4 h in the CO_2_ incubator prior to adding 100 µL of solubilization solution. After 16–18 h of incubation, the absorbance was recorded at 570 nm using a FLUOstar^®^ Omega microplate reader (BMG Labtech, Ortenberg, Germany). The well containing only medium and CDs without cells was served as a blank. After blank subtraction, the results were expressed as the percentage decrease in absorbance, relative to that of the untreated counterpart.

### 2.7. Evaluation of Preservative Effectiveness of CDs on Precooked Baby Clam During Refrigerated Storage

#### 2.7.1. Preparation of Precooked Edible Portion of Baby Clam Treated with CDs

The precooked edible portion of baby clams was treated with DF-CDs and RB-CDs at concentrations of 300 ppm and 600 ppm. A 30 g portion was placed into a laminated polynylon bag (12 × 15 cm^2^, 100 µm thickness), and the respective solutions with the known concentrations of CDs were sprayed with the exact minimum volumes to obtain the final CD concentration of precooked edible portions at 300 ppm and 600 ppm. The mixtures were mixed thoroughly within the bag. Control samples were treated with sterile distilled water in the same manner. All bags were then aseptically sealed using a sealing machine (MeC Impulse Sealer with Magnet, ME-300HIM, 500W (Impulse), Mercier Corporation, New Taipei, Taiwan). The sealed bags were stored in a refrigerator (Model SPA-0303A, Sanden Intercool, O.S.C. Cooling Company Limited, Bangkok, Thailand) at 4 ± 1 °C for 15 days. Analyses were conducted at 3-day intervals. For each analysis, samples were aseptically collected, while the remaining portions were kept on crushed ice.

#### 2.7.2. Microbiological Analyses

The total viable count (TVC) and psychrophilic bacterial count (PBC) of all samples were evaluated following the standard method outlined in the FDA’s Bacteriological Analytical Manual (BAM) as adapted by Palamae et al. [34]. Each sample (25 g) was mixed with 225 mL of 0.85% sterile saline. The mixture was homogenized at 300 rpm for 2 min using a Stomacher blender (M400, Seward, West Sussex, UK). Then, 1 mL of the homogenate was transferred into a test tube containing 9 mL of 0.85% sterile saline for serial dilution. From the diluted samples, 100 µL from each dilution was used for microbial enumeration using the spread plate method. The TVC and PBC were determined on the plate count agar (PCA, code: CM0463, Oxoid™, Thermo Fisher Scientific, Waltham, MA, USA) by incubating at 37 °C for 2 days and at 4 °C for 10 days, respectively.

#### 2.7.3. Chemical Analyses

The peroxide value (PV) and thiobarbituric acid reactive substance (TBARS) value were determined following the methods of Richards and Hultin [35] and Buege and Aust [36], respectively. For PV analysis, a sample (2 g) was homogenized with 22 mL of a methanol/chloroform mixture (1:2, *v*/*v*) at 13,500 rpm for 2 min. The homogenates were filtered and 2 mL of 0.5% NaCl solution was added to the resulting filtrate. The mixture was vortexed at moderate speed for 30 s and subsequently centrifuged at 3000× *g* for 3 min. The sample was separated into two distinct phases. From the lower phase, 2 mL was collected and was then mixed with 25 µL of 30% (*w*/*v*) ammonium thiocyanate and 25 µL of 20 mM FeCl_2_. The absorbance of the reaction mixture was recorded at 500 nm. Cumene hydroperoxide solutions (0–2 mg/L) served as the standard. The PV results were expressed in mg cumene hydroperoxide/100 g of sample.

For TBARS analysis, the samples (0.5 g) were homogenized with 2.5 mL of TBARS reagent containing TBA (0.0375 g/100 mL), TCA (15 g/100 mL) and HCl (0.25 M). The resulting mixture was heated at 95 °C for 10 min and then centrifuged at 3600× *g* for 20 min. Absorbance was measured at 532 nm. TBARS values were determined using a standard curve of malondialdehyde (MDA) ranging from 0 to 10 mg/L and expressed as mg MDA per 100 g of sample.

### 2.8. Statistical Analyses

A completely randomized design (CBD) was utilized throughout this study. All the experiments and analyses were performed in triplicate (*n* = 3). The data were expressed as the mean ± standard deviation. A one-way ANOVA was conducted, and Duncan’s multiple range test was applied to compare the means. Significant differences among the samples were considered at *p* ≤ 0.05. Data analysis was executed using the SPSS package (SPSS 27.0 for Windows, SPSS Inc., Chicago, IL, USA).

## 3. Results and Discussion

### 3.1. Characteristics of Synthesized Carbon Dots

#### 3.1.1. Visual Appearance and Color

**Figure 1:**
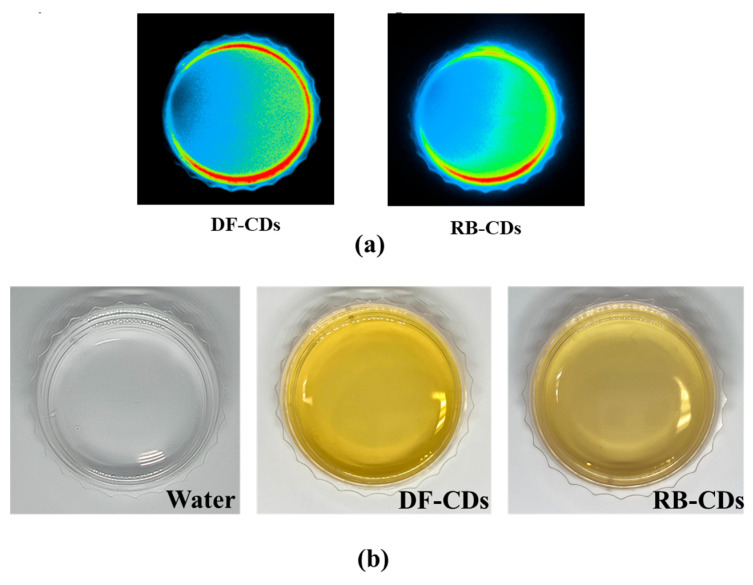
(**a**) Photographs of CDs under UV_365_ irradiation and (**b**) photographs of distilled water and CDs solutions under normal light. DF-CDs = dried chamomile flower carbon dots; RB-CDs = chamomile flower residual biomass carbon dots.

Both the DF-CDs and RB-CDs samples exhibited a yellowish-brown hue under normal light. In addition, the color was transformed to a bright blue when exposed to UV light, as shown in Figure 1a. This behavior is characteristic of CDs due to their inherent ability to emit light upon the exposure to UV light [37]. This emission is influenced by some factors such as particle size, surface functional groups and structural defects, etc., which directly impact the electronic band structure and fluorescence characteristics of the CDs [38]. Similar transformations in color under UV light have been reported for CDs synthesized from turmeric [37] and green tea [39], which turned blue under UV illumination. The observed color under UV light typically varies, based on the carbon precursor and the synthesis methods used for CDs preparation [37]. Color is directly linked to the intensity of fluorescence emitted when exposed to UV radiation.

The L* (lightness) values of DF-CDs and RB-CDs (Table 1) were recorded at 85.05 and 90.25, respectively. This indicated that the DF-CDs were slightly darker in color than the RB-CDs. The negative a* values (greenness) for DF-CDs and RB-CDs were −1.24 and −1.35, respectively, indicating that RB-CDs were slightly more greenish than the DF-CDs. Furthermore, the b* value (yellowness) of DF-CDs was 40.14, and that of RB-CDs was 33.94. A higher b* value of DF-CDs indicated a more yellowish color than that of RB-CDs, which could be visually observed from Figure 1b. Some pigments, phenolic compounds and essential oil in the flower might contribute to such a yellow color observed in the resulting CDs.

#### 3.1.2. UV-Vis Spectrophotometric and Photoluminescence Spectra

**Figure 2:**
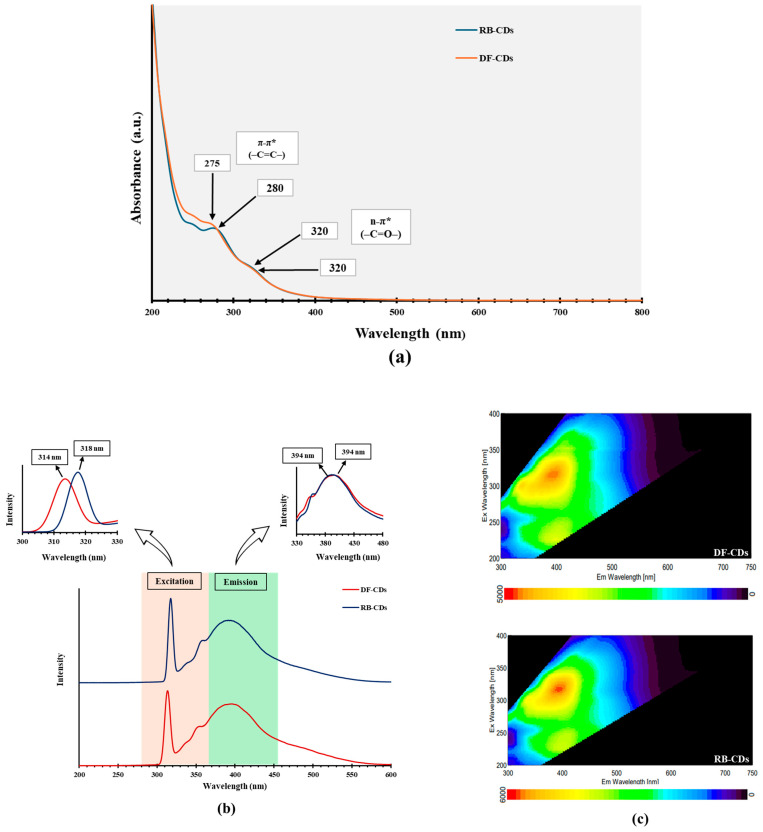
(**a**) UV –vis absorption spectrum, (**b**) fluorescence excitation and emission spectra and (**c**) 3D fluorescence responses at different excitation wavelengths from 200 to 400 nm and emission wavelength from 300 to 750 nm. DF-CDs = dried chamomile flower carbon dots; RB-CDs = chamomile flower residual biomass carbon dots.

UV-Vis absorption peaks were observed below 300 nm, and shoulder bands were obtained between 300 and 400 nm, similar to most synthesized CDs [25]. These phenomena are attributed to the π–π* transitions of –C=C– bonds in *sp^2^*-hybridized carbon domains or the n–π* transitions of –C=O– bonds [40]. These absorption peaks were followed by a tail extending into the visible range. CDs synthesized through different methods displayed absorption peaks at varying wavelengths or spectral regions [41]. The UV-Vis absorption spectra of both DF-CDs and RB-CDs (Figure 2a) showed almost similar absorption maxima in the UV region, which might be owing to the same sources of the CDs. DF-CDs exhibited an absorption maximum (λmax) at 275 nm due to π–π* (–C=C–) transitions, and a secondary peak was observed around 320 nm likely governed by n–π* (–C=O–).

Similarly, RB-CDs exhibited an absorption maximum (λmax) at 280 nm and a secondary peak at 320 nm. The peaks obtained are characteristic of the materials abundant in polyphenolic compounds naturally present in chamomile (e.g., apigenin, luteolin and flavonoids), which contain multiple conjugated double bonds and aromatic rings [42]. During carbonization or hydrothermal synthesis, these structures became embedded inside the CD’s core or remained as surface functional groups, thus still retaining their optical or electronic properties [43]. Similar UV-Vis peaks have been reported for CDs derived from other plant materials like green tea, turmeric, ginger, etc., likely resulting from residual aromatic compounds or the *sp^2^*-carbon core [44,45].

The photoluminescence spectra offered additional insights into the levels of energy related to emission bands [46]. The fluorescence spectra of DF-CDs and RB-CDs are depicted in Figure 2b, which present the emission peaks for the CDs derived from the dried chamomile flower and its residual biomass. The similar spectral peak positions of DF-CDs and RB-CDs suggested that both samples possessed a similar fluorescence behavior, which might be related to the similar structure and surface functional groups. Various functional groups such as hydroxyl (–OH), carboxyl (–COOH) and carbonyl (C=O) play a significant role in modulating photoluminescence excitation behavior [47]. By modifying the surface energy states of the material, key sites could be involved in radiative recombination. These altered surface states directly influenced the efficiency and characteristics of the photoluminescent emission. Both DF-CDs and RB-CDs exhibited almost similar emission and excitation peak characteristics. DF-CDs showed an emission peak at 394 nm with an excitation peak at 314 nm. Similarly, RB-CDs exhibited an emission peak at 394 nm with an excitation peak at 316 nm. The spectra of both CDs indicated their similarities in photoluminescent properties, suggesting that CDs derived from DF and RB exhibited comparable emission behavior. The fluorescence excitation and emission spectra for each type of CD are presented in a 3D fluorescence map (Figure 2c). The CDs exhibited a progressive blue shift in their emission peaks as the excitation wavelength increased from 320 to 460 nm, indicating their adjustable luminescence behavior. An upsurge in photoluminescence excitation intensity was also observed, reflecting stronger light absorption and improved emission efficiency. This behavior implied that both CDs possessed uniform emission sites across their surface, more likely caused by the presence of abundant surface functional groups [10].

#### 3.1.3. UV Blocking Property

**Figure 3:**
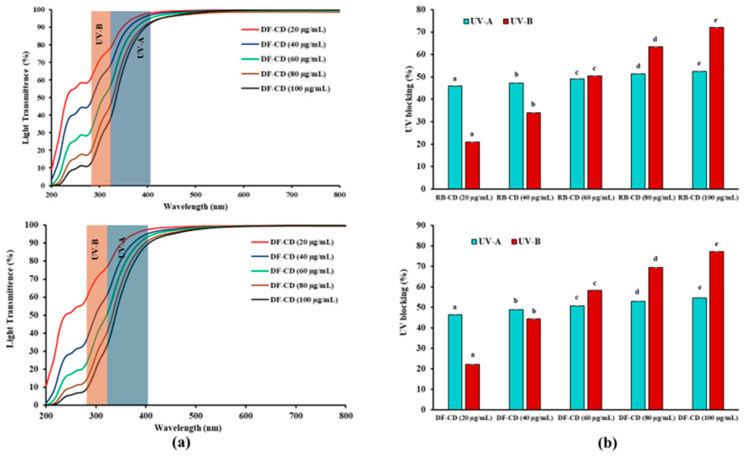
(**a**) The UV–visible light transmittance spectra and (**b**) %UV-A (320–400 nm) and %UV-B (280–320 nm) blocking properties of DF-CDs and RB-CDs at various concentrations (20–100 µg/mL). The bars represent the mean ± SD (*n* = 3). Different lowercase letters on the bar within the same UV light range denote a significant difference (*p* < 0.05). DF-CDs = dried chamomile flower carbon dots; RB-CDs = chamomile flower residual biomass carbon dots.

The UV-vis light transmittance profiles and UV light blocking capabilities of DF-CDs and RB-CDs at concentrations ranging from 20 to 100 μg/mL are presented in Figure 3a,b.

These CDs exhibited strong scattering and absorption characteristics, enabling them to effectively convert UV photons into heat [48]. The light transmittance of DF-CDs and RB-CDs was remarkably decreased with the changing concentrations of both CDs. Solutions of DF-CDs and RB-CDs at different concentrations showed the reduced light transmittance within the UV range (200–400 nm), indicating their ability to absorb UV radiation. Both the CD solutions had complete (100%) transmittance in the visible light region, while in the UV range of 200–400 nm, the transmittance dropped sharply (*p* < 0.05). The UV-A and UV-B shielding efficiency of both DF-CDs and RB-CDs was improved with enhanced concentrations. For concentrations ranging from 20 to 100 µg/mL, DF-CDs inhibited UV-A and UV-B radiation by 46.33–54.67% and 22.17–77.15%, respectively (*p* < 0.05). Similarly, RB-CDs exhibited UV-A and UV-B blocking capacities of 46.01–52.54% and 22.17–77.15%, respectively. The highest UV blocking capacity of both DF-CDs and RB-CDs towards UV-A and UV-B was recorded at 100 µg/mL. Plant- or flower-derived CDs showed strong UV absorption. When they were embedded into films or blend films, the effective UV blocking property was achieved while retaining visible transparency. This characteristic makes them suitable agents for sunscreens, cosmetics and UV-protective food packaging [49].

#### 3.1.4. TEM and Particle Size

**Figure 4:**
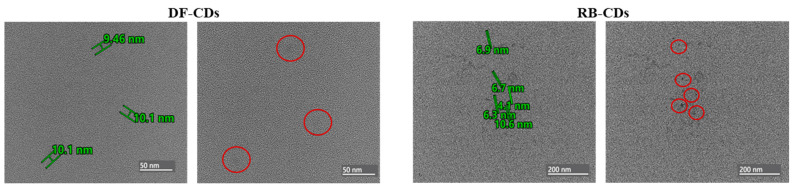
TEM images of DF-CDs and RB-CDs. The red circles mark the indentified CDs. DF-CDs = dried chamomile flower carbon dots; RB-CDs = chamomile flower residual biomass carbon dots.

TEM images offered detailed information about the morphology and size distribution of the CDs obtained from DF and RB. The size and morphology of the synthesized CDs were analyzed using TEM, as shown in Figure 4. The DF-CDs exhibited particle sizes ranging from 9.46 to 10.60 nm, with an average diameter of 10.05 ± 0.47 nm. Likewise, the RB-CDs showed particle sizes between 4.10 and 10.06 nm, with an average diameter of 7.00 ± 2.70 nm (Figure 4). TEM images showed that the CDs exhibited a spherical morphology, which aligned well with the quantum dot size range documented in the previous studies [50]. The size of CDs was varied, depending on the precursor materials and the synthesis techniques employed. CDs generally exhibit sizes ranging from 2 to 10 nm, depending on the sources. CDs from agricultural waste materials, including fruit peels and crop residues, have been reported to have a nano size [51].

#### 3.1.5. FTIR Spectra

**Figure 5:**
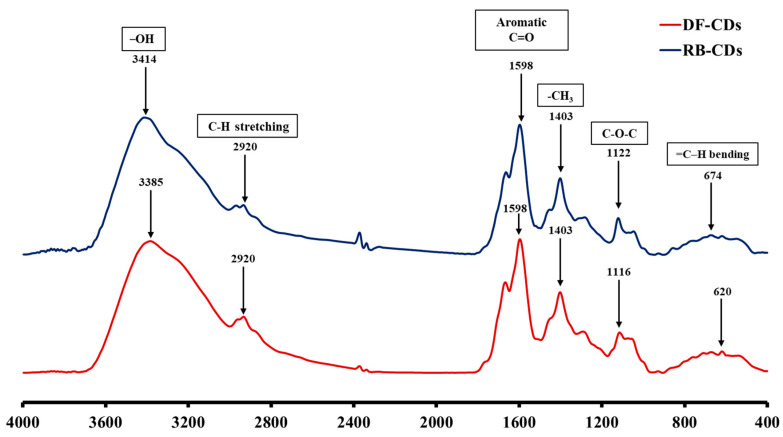
FTIR spectra of DF-CDs and RB-CDs. DF-CDs = dried chamomile flower carbon dots; RB-CDs = chamomile flower residual biomass carbon dots.

Progression during the hydrothermal reaction of the CDs was monitored by FTIR spectra, which are able to reveal the functional groups present on the surface of the CDs. The FTIR spectra of DF-CDs and RB-CDs are depicted in Figure 5. Wide absorption band at 3414 cm^−1^ (RB-CDs) and 3385 cm^−1^ (DF-CDs) are attributed to the stretching vibrations of –OH groups, whereas the peaks observed at 1598 cm^−1^ for both RB-CDs and DF-CDs are linked to the stretching vibrations of aromatic carbonyl (C=O) groups, typically associated with –COOH [40,43]. The peaks observed at 2920 cm^−1^ for both DF-CDs and RB-CDs were attributed to the asymmetric and symmetric C–H stretching vibrations of the aliphatic chains [52]. The antisymmetric in-plane bending vibration of the CH_3_ group was observed at 1404 cm^−1^ for both RB-CDs and DF-CDs. The peaks found at 1122 cm^−1^ for RB-CDs and 1116 cm^−1^ for DF-CDs corresponded to the stretching vibrations of the C–O–C bond [53]. The characteristic peaks observed at 674 cm^−1^ and 620 cm^−1^ for RB-CDs and DF-CDs, respectively, indicated the presence of =C–H bending vibrations [54]. FTIR analysis confirmed the presence of phenolic hydroxyl groups in RB-CDs and DF-CDs, highlighting the role of polyphenols in their characteristics and their bioactivities, especially the remaining OH groups.

### 3.2. Antioxidant Activities

CDs have attracted considerable interest due to their exceptional antioxidant activity, which is mainly linked to their plentiful surface functional groups such as –OH, C=O, etc., capable of scavenging free radicals and reactive oxygen species [55]. The antioxidant activities of DF-CDs and RB-CDs at a concentration of 1000 μg/mL are presented in Table 2. DF-CDs demonstrated significantly higher antioxidant activities than those of RB-CDs in all assays (*p* < 0.05). DF-CDs exhibited a DPPH-RSA level of 56.42 µmol TE/mL, while RB-CDs had an activity level of 54.60 µmol TE/mL. When tested using the ABTS-RSA assay, DF-CDs achieved 95.51 µmol TE/mL, whereas RB-CDs showed 91.77 µmol TE/mL. DF-CDs exhibited a FRAP of 136.47 µmol TE/mL, which was slightly higher than the FRAP of RB-CDs (130.98 µmol TE/mL). MCA marginally followed a similar trend, with DF-CDs having 16.46 µmol TE/mL and RB-CDs possessing 14.68 µmol EE/mL.

The higher antioxidant activities of DF-CDs across all assays than those of RB-CDs indicated that dried flowers could serve as a better potential precursor, which could yield more bioactive compounds with favorable surface functionalities than the residual biomass (RB) obtained after essential oil extraction. Polar leaf extracts of *Vernonia amygdalina* also showed superior DPPH-RSA and ABTS-RSA, due to a greater abundance of electron- or hydrogen-donating groups, likely associated with intact phenolic and flavonoid constituents [56]. The FRAP results further supported the strong reducing capacity of DF-CDs, reflecting their efficiency in electron transfer processes [57]. The enhanced MCA also highlighted the stronger ability of DF-CDs to chelate pro-oxidant metal ions such as Fe, thus potentially mitigating metal-induced oxidative stress [58]. The lower activities observed in RB-CDs might result from the degradation or removal of antioxidant phytochemicals during the extraction of essential oil. Essential oil, which was extracted, has been documented to have strong antioxidant activity [59]. Therefore, RB contained a lower content of those active components. These findings emphasized that the precursor of CDs played a crucial role in determining the antioxidant capacity of CDs. Overall, DF-CDs were more effective in terms of antioxidant activity for applications requiring potent free radical scavenging and metal chelating properties.

### 3.3. Antimicrobial Activities

#### 3.3.1. MIC and MBC

The MIC and MBC of CDs synthesized from DF and RB were evaluated against two foodborne pathogenic bacterial strains (*Escherichia coli* and *Listeria monocytogenes*) and two spoilage bacteria (*Shewanella putrefaciens* and *Pseudomonas aeruginosa*), as summarized in Table 3. Both DF-CDs and RB-CDs exhibited antibacterial activity across all tested strains, including both Gram-positive and Gram-negative strains, with MIC values ranging from 2 to 8 mg/mL. Notably, *Listeria monocytogenes*, a Gram-positive bacterium, was the most susceptible strain, with the lowest MIC of 2 mg/mL for both types of CDs. In contrast, *Escherichia coli* and *Shewanella putrefaciens*, Gram-negative, required higher concentrations (8 mg/mL) to inhibit growth. Basically, Gram-negative strains have a thin peptidoglycan layer, but they contain a lipopolysaccharide outer membrane [60]. As a result, the Gram-negative strains were more resistant to the CDs tested. The MBC values had a similar trend, in which CDs showed bactericidal effect toward *Listeria monocytogenes* at 16 mg/mL, despite its low MIC. This suggested a bacteriostatic effect at lower concentrations and a requirement for higher doses to achieve complete bacterial eradication. *Escherichia coli* also exhibited a high MBC of 16 mg/mL, consistent with its known resilience to antimicrobial agents [61]. For *Pseudomonas aeruginosa* and *Shewanella putrefaciens*, MBC values were 8 mg/mL, indicating effective bactericidal activity at relatively low levels. *Pseudomonas* spp. and *Shewanella* spp. have been known to be the major contributors of spoilage in seafoods [62]. Moreover, no significant differences were observed between DF-CDs and RB-CDs in terms of MIC and MBC values, suggesting that the antimicrobial efficacy of CDs was not influenced by the precursors used, namely chamomile flower or residual biomass.

CDs can potentially induce bacterial cell oxidative stress through mechanisms such as disruption, lipid interaction, ROS generation and electrostatic interactions. These processes may damage vital cellular components [51,63]. Furthermore, CDs may assemble with the lipid bilayer of microbial membranes, leading to structural modifications that weaken membrane integrity and cause the leakage of intracellular contents [64]. When CDs enter the bacterial cell through the membrane, they cause protein damage and lead to the leakage of internal cytoplasmic contents. This results in chromosome condensation and the disruption of DNA replication [65]. Therefore, both DF-CDs and RB-CDs could be used as antimicrobial agents to prolong the shelf life of seafood by inactivating spoilage bacteria.

Moreover, the MBC values of both CDs (8–16 mg/mL) were relatively high, which might limit direct application in real food systems. Such high values suggested that their use at high concentrations could raise concerns related to cost, sensory quality and regulatory acceptance. To overcome this obstacle, the use of these CDs in combination with other preservation methods (e.g., mild heat, high-pressure processing, cold plasma, modified atmosphere packaging or natural preservatives) could be a means to achieve a hurdle effect. As a consequence, the preservation efficacy could be enhanced, while CDs were used at a low level.

In the present study, the antibacterial activity of CDs was investigated using the resazurin dye assay, under the standard incubation conditions in the absence of visible light irradiation. This approach was taken to evaluate the intrinsic antibacterial potential of the CDs, regardless of any photocatalytic or photo-activated mechanisms. It is recognized that the exposure to visible light may enhance or change the antibacterial responses in certain systems [66,67], but this parameter was not included in the present investigation. Nevertheless, the influence of visible light on antibacterial activity should be considered in future research to elucidate its role in the antibacterial efficacy of CDs.

#### 3.3.2. Bacterial Growth and Biofilm Formation

**Figure 6:**
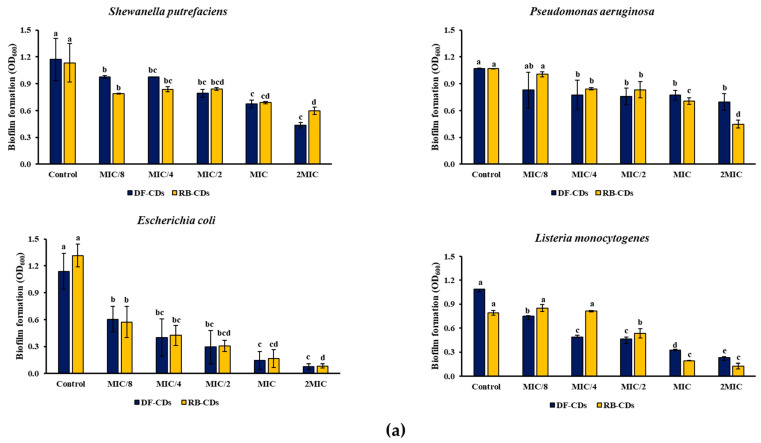

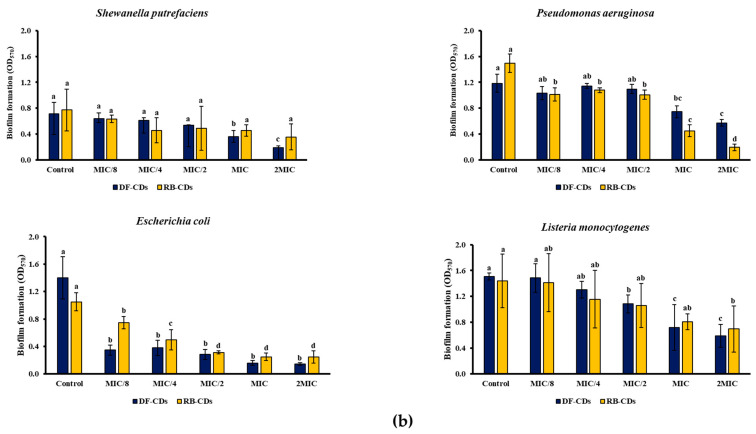
(**a**) The inhibition of the growth and (**b**) biofilm formation of *Shewanella putrefaciens*, *Pseudomonas aeruginosa*, *Escherichia coli* and *Listeria monocytogenes* after treatment with DF-CDs and RB-CDs at different concentrations. The bars represent the mean ± SD (*n* = 3). Different lowercase letters on the bar within the same type of CDs denote a significant difference (*p* < 0.05). DF-CDs = dried chamomile flower carbon dots; RB-CDs = chamomile flower residual biomass carbon dots.

Bacterial growth in the presence of CDs synthesized from DF and RB at different concentrations (MIC/8 to 2 MIC) was assessed against two foodborne pathogenic bacterial strains (*Escherichia coli* and *Listeria monocytogenes*) and two spoilage bacteria (*Shewanella putrefaciens* and *Pseudomonas aeruginosa*) as monitored by the decrease in OD_600_ (Figure 6a). Both DF-CDs and RB-CDs demonstrated dose-dependent inhibition of bacterial growth. The most prominent inhibition effects were observed at the MIC and 2MIC levels. For *Listeria monocytogenes* and *Escherichia coli*, the OD_600_ of DF-CDs was reduced from 1.086 and 1.140 (control) to 0.232 and 0.078 (2MIC), respectively, after 48 h of incubation, while RB-CDs showed similar reductions when compared to the control. On the other hand, *Shewanella putrefaciens* and *Pseudomonas aeruginosa* exhibited moderate resistance toward both CDs. For *Shewanella putrefaciens* and *Pseudomonas aeruginosa*, the OD_600_ of DF-CDs was reduced from 1.173 and 1.070 (control) to 0.436 and 0.694 (2MIC), and the OD_600_ of RB-CDs decreased from 1.133 and 1.067 (control) to 0.598 and 0.448 (2MIC), respectively, after 48 h of incubation. The results suggested the strong antibacterial efficacy of both CDs. These differences in inhibition might reflect the variations in the bacterial cell wall composition of the varying bacteria tested. The physicochemical properties of both CDs, involving surface charge and functional groups, could also affect the cell membrane of different bacteria in varying fashions [68].

DF-CDs reduced the formation of *Shewanella putrefaciens* biofilm as indicated by the decrease in OD_570_ from 0.713 to 0.190, while RB-CDs achieved comparable reductions from 0.773 to 0.355 (Figure 6b). For *Pseudomonas aeruginosa*, RB-CDs were more effective than DF-CDs, by reducing OD_570_ from 1.496 to 0.195, while DF-CDs reduced OD_570_ from 1.184 to 0.573. Moreover, in the case of *Listeria monocytogenes* and *Escherichia coli*, DF-CDs were more effective than RB-CDs in inhibiting the formation of biofilm. DF-CDs reduced the OD_570_ of the control from 1.508 to 0.588 and from 1.399 to 0.142 at the 2MIC level in the case of *Listeria monocytogenes* and *Escherichia coli*, respectively.

Overall, both DF-CDs and RB-CDs exhibited a broad spectrum inhibition towards bacteria and also showed anti-biofilm activity. Overall, DF-CDs showed greater efficacy against Gram-positive strains, and RB-CDs performed better against certain Gram-negative bacteria. This might be due to the quantum-sized characteristics (Figure 4) of CDs, thus favoring transportation through bacterial cell walls. However, efficacy might be varied depending on the cell walls of various bacteria, which showed different degrees of resistance to the CDs used. In addition, they were able to suppress certain enzyme functions required for bacterial growth [69]. Consequently, this process results in cell membrane rupture, elevated oxidative stress and ultimately bacterial cell death [66]. The ability to inhibit biofilm formation even at sub-MICs suggested the potential of CDs from both sources to be used for seafood preservation. These findings highlighted the potential of DF-CDs and RB-CDs as eco-friendly antimicrobial agents in foods and therapeutic applications.

### 3.4. Cytotoxicity of Synthesized CDs

**Figure 7:**
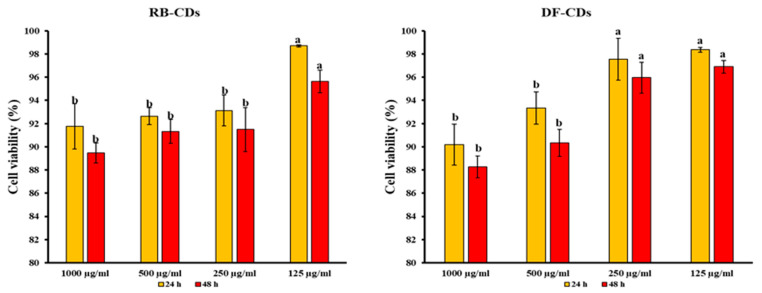
The cell viability of BJ cells after treatment with DF-CDs and RB-CDs at various concentrations for 24 h and 48 h. The bars represent the mean ± SD (*n* = 3). Different lowercase letters on the bar within the same incubation time denote a significant difference (*p* < 0.05). DF-CDs = dried chamomile flower carbon dots; RB-CDs = chamomile flower residual biomass carbon dots.

The cytotoxicity of DF-CDs and RB-CDs at concentrations of 125, 250, 500 and 1000 μg/mL toward the BJ human fibroblast cell line was evaluated using the MTT assay over 24 and 48 h. The results are presented in Figure 7. For DF-CDs, cell viability remained above 90% at all concentrations after 24 h of incubation, with the highest viability observed at 125 μg/mL (98.36%) and the lowest viability at 1000 μg/mL (90.17%). After 48 h, a slight reduction in viability was noted, ranging from 88.26% at 1000 μg/mL to 96.89% at 125 μg/mL. Similarly, RB-CDs demonstrated high cell viability, with values ranging from 91.76% to 98.96% after 24 h. The highest viability was recorded at 125 μg/mL, while the lowest was obtained at 1000 μg/mL. At 48 h, cell viability remained above 89% across all concentrations, with the highest value observed at the concentration of 125 μg/mL (95.63%). 

Overall, both DF-CDs and RB-CDs showed excellent cytocompatibility, as evidenced by high cell viability, which consistently exceeded 88% even at the highest concentration and longest exposure time. A slight decrease in viability was observed, particularly at 1000 μg/mL, which was consistent with typical cellular responses to nanoparticle exposure [70,71]. The dose-dependent decline in viability was very low, indicating that prolonged exposure did not significantly compromise cell survival. Some differences in cell viability between the two CDs might be attributed to variations in surface chemistry, particle size, or functional groups between the two types of CDs, which could be observed from the TEM and FTIR results of both CDs. The high biocompatibility of both DF-CDs and RB-CDs and those in other studies underscores the potential of CDs for biomedical applications, food preservation, drug delivery, bioimaging and therapeutic interventions [52,63,72].

### 3.5. Use of CDs as Preservative of Precooked Baby Clam Stored at Refrigerated Temperature

#### 3.5.1. TVC and PBC

**Figure 8:**
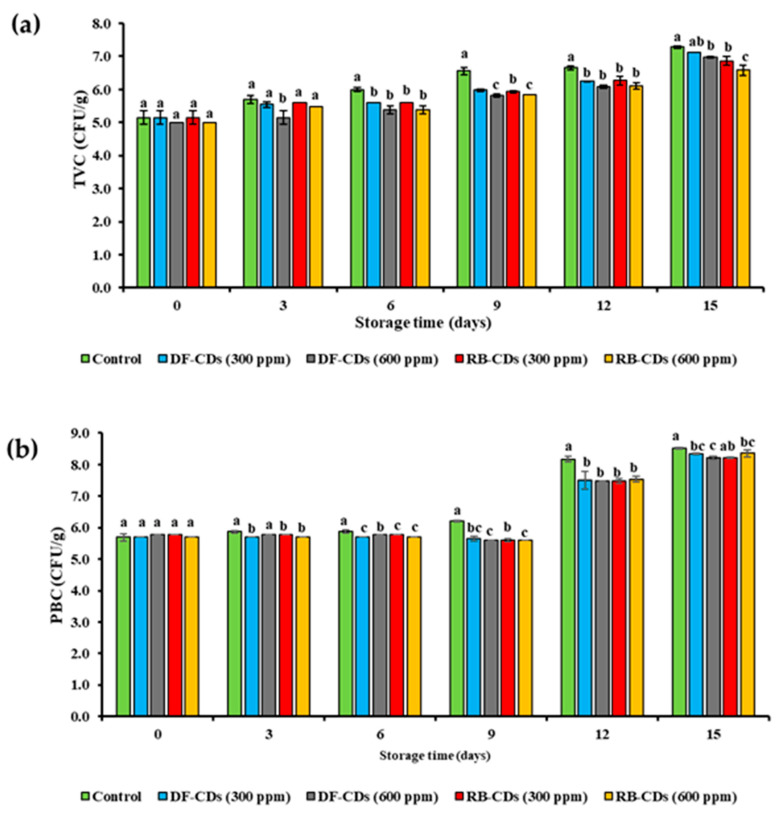
(**a**) The total viable count and (**b**) psychrophilic bacteria count of PBC-EP after treatment with DF-CDs and RB-CDs at different concentrations (300 ppm and 600 ppm) for 15 days. The bars represent the mean ± SD (*n* = 3). Different lowercase letters on the bar within the same storage time denote a significant difference (*p* < 0.05). DF-CDs = dried chamomile flower carbon dots; RB-CDs = chamomile flower residual biomass carbon dots.

The changes in the TVC of the treated and control PBC-EP samples during 15 days of refrigerated storage are presented in Figure 8a. Across all treatments, a gradual increase in microbial load was observed as storage time increased. Nonetheless, the rate of increase varied notably between treatments.

On day 0, the initial TVC values for all groups were 5.0 to 5.2 log CFU/g, indicating high microbial load prior to storage. During shucking, the edible portion obtained became more exposed to external environment such as utensils, etc., particularly during cooling and packing. In addition, the edible portion of bivalves is susceptible to spoilage mainly due to the presence of a high amount of protein and amino acids [73]. However, the load was still under the microbial limit for consumption (6 log CFU/g). As storage progressed, the control group exhibited the highest and most rapid increase (5.2 to 7.3 CFU/g) after 6 days of storage, surpassing the acceptable microbial limit. Thus, the control could be suitable for consumption after storage for 3 days. In contrast, samples treated with DF-CDs and RB-CDs at both 300 ppm and 600 ppm maintained a significantly lower TVC until day 9, at which the TVC was still below the permissible microbial load. The TVC was above the limit when the samples were stored for 12 days, regardless of the types and concentration of CDs used.

In general, RB-CDs demonstrated slightly lower efficacy in the reduction in microbial growth than DF-CDs. According to the International Commission on Microbiological Specifications for Foods (ICMSF), the maximum permissible level for the total viable count (TVC) in fresh aquatic products is 6 log CFU/g [74]. In the present study, the initial microbial load in the control sample was approximately 5 log CFU/g, which was still below this threshold. Such microbial levels in filter feeders (e.g., mussels, clams, scallops) are not uncommon. In addition, the microbiological quality of the surrounding water and hygienic handling practices determine the microbial load of samples [75]. The results indicated that CDs effectively reduced microbial loads and retarded the microbial growth of samples with initially high microbial loads. Such findings are useful for bivalves and related seafood industries, where raw material quality can fluctuate, and robust preservation strategies are needed.

The PBCs of the control and the treated PBC-EP samples during 15 days of refrigerated storage are shown in Figure 8b. At day 0, all samples displayed similar counts, indicating uniform starting microbial loads. Over the storage period, a progressive increase in the PBC was observed in all groups, consistent with the growth of cold-tolerant microorganisms under refrigeration. Palamae et al. [34] found that the major bacteria contributing to the spoilage of PBC-EP included *Shewanella* spp., *Pseudomonas* spp., etc. The control samples exhibited the most rapid and continuous rise in the PBC, reaching the highest levels by day 15. In contrast, DF-CD-treated samples, especially at 600 ppm, maintained the lowest bacterial counts throughout storage. A significantly lower PBC in CD-treated samples (*p* < 0.05) in comparison to the control was found from day 6 onwards. RB-CD-treated samples also demonstrated a notable reduction in the PBC relative to the control, though the inhibitory effect was consistently lower than that observed for DF-CDs. This difference in efficacy might be attributed to variations in particle size, surface chemistry and functional groups between the two CDs used for sample treatment. This potential reduction in the TVC as well as the PBC was likely due to the presence of abundant surface functional groups and reactive oxygen species generation by the CDs, which disrupt bacterial cell membranes and impair metabolic functions [76]. In addition, high-pressure processing (HPP) has been used to prolong the shelf life of PBC-EP. Palamae et al. [34] applied HPP for the treatment of PBC-EP at 400 MPa for 4 min. It was found that the sample could be kept up to 12 days at refrigerated temperature. Therefore, the combination of CDs and HPP could be a means to increase their potential to be used for the inactivation of microorganisms, especially spoilage bacteria.

#### 3.5.2. TBARS and PV

**Figure 9:**
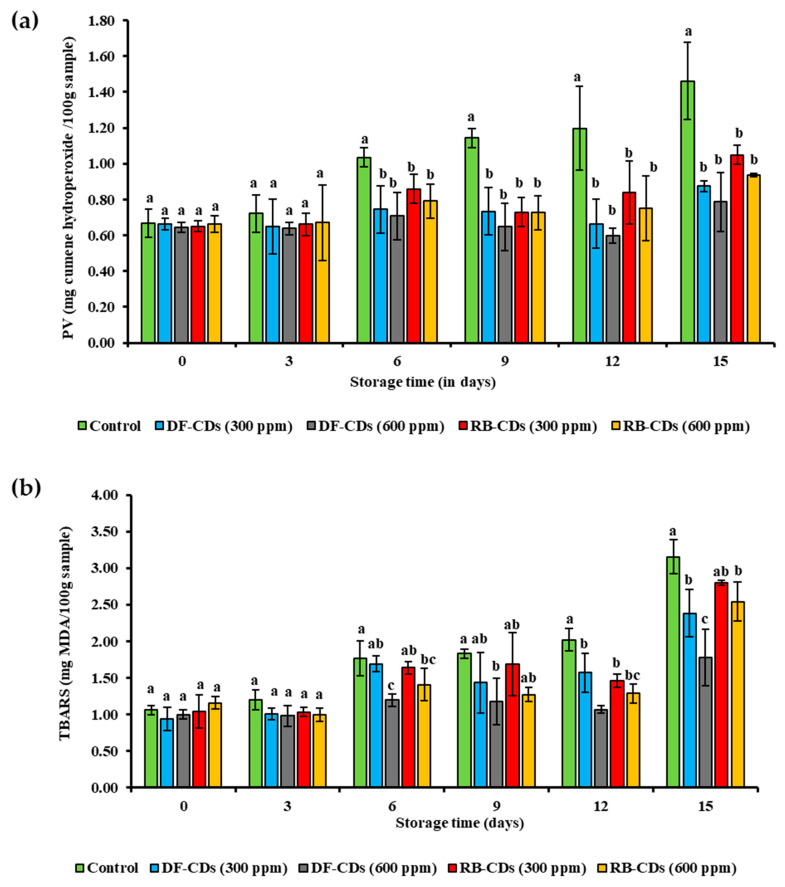
(**a**) The peroxide value and (**b**) TBARS value of PBC-EP treated with DF-CDs and RB-CDs at different concentrations (300 ppm and 600 ppm) during storage for 15 days. The bars represent the mean ± SD (*n* = 3). Different lowercase letters on the bar within the same storage time denote a significant difference (*p* < 0.05). DF-CDs = dried chamomile flower carbon dots; RB-CDs = chamomile flower residual biomass carbon dots.

The effectiveness of treatments with DF-CDs and RB-CDs in inhibiting the lipid oxidation of PBC-EP samples was evaluated by the PV and TBARS value over a 15-day storage period. Figure 9a (PV) shows the formation of primary oxidation products (hydroperoxides), while Figure 9b (TBARS) represents the secondary oxidation products (malondialdehyde). All samples, including the control and all treated groups (both CDs at 300 ppm and 600 ppm), showed similar low PVs (0.6–0.7 mg cumene hydroperoxide/100 g sample) and TBARS values (around 1.0 mg MDA/100 g sample) at day 0, indicating that a low level of lipid oxidation took place in PBC-EP at day 0. During storage, the control group exhibited a continuous and significant increase (*p* < 0.05) in both the PV and TBARS value. The PV for the control reached a maximum of 1.4 mg cumene hydroperoxide/100 g sample by day 15, and the TBARS value exceeded 3.0 mg MDA/100 g sample at the end of storage. This demonstrated that PBC-EP was highly susceptible to oxidative deterioration under refrigerated conditions. In contrast, all treatment using CDs (300 ppm), DF-CDs (600 ppm) and RB-CDs (300 ppm), and RB-CDs (600 ppm) effectively suppressed the rise in the PV and TBARS value of the sample throughout the 15 days.

The antioxidant efficacy of both CDs increased in a dose-dependent manner. CDs at the higher concentration (600 ppm) resulted in lower PVs and TBARS values of PBC-EP samples, compared to the lower concentration (300 ppm) for both CDs used. On day 15, the PV of the RB-CDs (600 ppm) sample was 0.9 mg cumene hydroperoxide/100 g sample, whereas the PV of the RB-CDs (300 ppm) sample was 1.05 mg cumene hydroperoxide/100 g sample. A similar trend was observed for TBARS, where the samples treated with both CDs at 600 ppm maintained lower values than those treated with CDs at 300 ppm. Overall, both DF-CDs and RB-CDs were effective in reducing lipid oxidation. However, there was no significant difference in their performance at the same concentration. The PVs and TBARS values for the DF (300 ppm) samples were similar to those of the RB (300 ppm) sample. The same trend was observed for both CDs at 600 ppm. This suggested that both CDs possessed comparable antioxidant properties in PBC-EP. The PV results, representing the initial stages of lipid oxidation, showed that both CDs were able to delay the formation of hydroperoxides [77]. The TBARS results indicated that both CDs also successfully inhibited the formation of secondary oxidation products associated with the off-odor and off-flavor in the samples [78]. DF-CDs and RB-CDs were demonstrated to exhibit radical scavenging activity and reducing power in an in vitro study (Table 2). The radical scavenging capacity of antioxidants has been known to play a key role in the retardation of lipid oxidation in seafoods [79]. In addition, metal chelation was involved in the lower lipid oxidation levels in bivalve mollusk [80]. Therefore, both CDs were effective in mitigating lipid oxidation, and their efficacy was directly correlated with the concentrations of them used.

## 4. Conclusions

CDs were successfully synthesized from dried German chamomile flowers (DF) and their residual biomass (RB). Both DF-CDs and RB-CDs with a nano size exhibited strong antioxidant and antimicrobial properties, in a dose-dependent fashion. They possessed an ability to scavenge free radicals and showed reducing power. They were able to inhibit the growth of various spoilage and pathogenic bacteria, including *Shewanella putrefaciens*, *Pseudomonas aeruginosa*, *Escherichia coli* and *Listeria monocytogenes*. Furthermore, the CDs were found to be low-cytotoxic in nature. Both DF-CDs and RB-CDs at 600 ppm could lengthen the shelf life of the precooked baby clam edible portion during refrigerated storage up to 12 days. Apart from the retardation of microbial growth, they could lower the lipid oxidation level of the sample during storage. Overall, chamomile flower and its residual biomass could be used to produce multifunctional CDs, which can serve as a novel and natural alternative to synthetic preservatives in seafoods, including bivalves.

## Figures and Tables

**Table 1:** L*, a* and b* values of carbon dots synthesized from dried German chamomile flower and residual biomass.

Sample Name	L*	a*	b*
DF-CDs	85.05 ± 0.25	−1.24 ± 0.02	40.14 ± 0.06
RB-CDs	90.25 ± 0.16	−1.35 ± 0.03	33.94 ± 0.11

Values are presented as mean ± SD (*n* = 3). DF-CDs: dried chamomile flower carbon dots; RB-CDs: chamomile flower residual biomass carbon dots.

**Table 2:** Antioxidant activities of carbon dots synthesized from dried German chamomile flower and residual biomass at concentration of 1000 µg/mL.

Sample Name	DPPH-RSA (µmol TE/mL)	ABTS-RSA (µmol TE/mL)	FRAP (µmol TE/mL)	MCA (µmol EE/mL)
DF-CDs	56.42 ± 0.60 ^a^	95.51± 5.38 ^a^	136.47 ± 3.79 ^a^	16.46 ± 2.75 ^a^
RB-CDs	54.60 ± 1.16 ^a^	91.77 ± 9.16 ^b^	130.98 ± 1.02 ^b^	14.68 ± 1.72 ^a^

Values are presented as mean ± SD (*n* = 3). Different lowercase superscripts in same column indicate significant differences (*p* < 0.05). DPPH-RSA: DPPH radical scavenging activity; ABTS-RSA: ABTS radical scavenging activity; FRAP: ferric reducing antioxidant power; MCA: metal chelating activity. DF-CDs: dried chamomile flower carbon dots; RB-CDs: chamomile flower residual biomass carbon dots; TE: Trolox equivalent; EE: EDTA equivalent.

**Table 3:** MIC and MBC of carbon dots synthesized from dried German chamomile flower and residual biomass.

Sample Name	MIC		MBC	
	DF-CDs	RB-CDs	DF-CDs	RB-CDs
*Shewanella putrefaciens*	8 mg/mL	8 mg/mL	8 mg/mL	8 mg/mL
*Pseudomonas aeruginosa*	4 mg/mL	4 mg/mL	8 mg/mL	8 mg/mL
*Escherichia coli*	8 mg/mL	8 mg/mL	16 mg/mL	16 mg/mL
*Listeria monocytogenes*	2 mg/mL	2 mg/mL	16 mg/mL	16 mg/mL

DF-CDs: dried chamomile flower carbon dots; RB-CDs: chamomile flower residual biomass carbon dots.

## Data Availability

The original contributions presented in this study are included in the article; further inquiries can be directed to the corresponding author.
